# Construction of a sensory quality evaluation model for tobacco leaves from Henan Province and its application in tobacco quality assessment

**DOI:** 10.3389/fpls.2026.1792794

**Published:** 2026-05-11

**Authors:** Xiujuan Xu, Longhe Wang, Jun Hu, Xiaoyuan Tian, Qingzhao Shi, Kai Cui, Xutao Li, Weichen Zhang, Shuoye Zhou, Wenfen Zhang, Guangqing Chen, Chunqiang Yang

**Affiliations:** 1 Zhengzhou Tobacco Research Institute of National Tobacco Corporation, Zhengzhou, China; 2 Henan Tobacco Company, Zhengzhou, China; 3 College of Chemistry, Zhengzhou University, Science Avenue, Zhengzhou, Henan, China

**Keywords:** agronomic regulation, aroma components, multivariate statistical analysis, sensory quality evaluation, tobacco leaves

## Abstract

This study was designed to establish a sensory-oriented quality evaluation model for tobacco leaves, systematically elucidate the key chemical basis underlying the sensory attributes of tobacco from representative growing regions in Henan Province, and identify the key aroma components influencing their quality. A total of 47 tobacco leaf samples were collected from three major producing areas in Henan Province, namely Pingdingshan, Sanmenxia, and Nanyang. Professional sensory evaluation and gas chromatography–mass spectrometry (GC–MS) were employed to quantitatively analyze 29 chemical constituents, including nicotine. Multivariate statistical approaches, such as principal component analysis (PCA) and correlation analysis, were applied to construct the sensory quality evaluation model and identify key aroma components. Furthermore, the effects of different agronomic factors (fertilization, harvesting methods, plant growth vigor, and single-leaf weight) on these key components were investigated. PCA revealed that the first two principal components cumulatively accounted for 52% of the total variance, representing sweet/fruity and green/woody characteristics, respectively. Correlation analysis identified 13 key aroma components significantly associated with sensory quality, including 3-hydroxy-2-butanone, benzaldehyde, linalool, *β*-damascone, and megastigmatrienone, among others. The established sensory–chemical correlation model effectively evaluated and predicted the sensory quality of tobacco leaves, revealing intrinsic relationships between key aroma components and sensory attributes. It is important to note that the samples utilized in this research are exclusively from Henan Province; therefore, caution should be exercised when generalizing these findings to tobacco leaves from other geographical regions or genetic contexts. The findings provide a scientific basis for enhancing the sensory comfort and industrial usability of tobacco leaves through precise agronomic regulation, such as optimized fertilization and harvesting practices.

## 1 Introduction

As one of the three major origins of flue-cured tobacco in China, Henan Province is renowned for its tobacco leaves with rich aroma, full smoke volume, and excellent blendability, making them an indispensable component in domestic cigarette formulations (Cui et al., 2018). The province exhibits significant ecological diversity across its tobacco-growing regions, which are broadly categorized into four production areas: Central Henan (Pingdingshan, Xuchang, Luohe), Western Henan (Sanmenxia, Luoyang), Southern Henan (Nanyang, Zhumadian, Xinyang), and Eastern Henan (Zhoukou, Shangqiu). Due to spatial variations in soil types, topography, and climatic conditions, tobacco leaves from these regions display distinct differences in chemical composition and sensory profiles (Kim and An, 2020; Tang et al., 2020; Cui et al., 2023). While this diversity provides a resource base for developing characteristic tobacco styles, it also poses challenges for quality consistency control.

Although the overall quality of Henan tobacco remains stable, several persistent issues limit its application in premium cigarette blends (Teng et al., 2024). These include excessively high single-leaf weight and leaf density in some regions, which adversely affect filling power and combustion performance; insufficient maturity in upper leaves, leading to tight leaf structure and poor permeability (Wu et al., 2024); and undesirable sensory attributes such as heavy off-odor, lingering aftertaste, and noticeable irritation, which compromise smoking purity and satisfaction (Zhao et al., 2023). These sensory shortcomings are fundamentally attributed to imbalances in the composition and coordination of complex chemical constituents within the tobacco leaves.

The sensory quality of tobacco is the ultimate perceptual manifestation of its internal chemical composition. Thousands of compounds collectively form the material basis that determines the leaf’s fragrance characteristics, smoke properties, and flavor profile (Liu et al., 2022; Jiang et al., 2024). Therefore, precisely deciphering the causal relationships between chemical composition and sensory quality is a scientific prerequisite for targeted quality improvement (Hao et al., 2024). Conventional chemical analyses often remain at the level of “content determination” and “statistical correlation,” falling short of directly identifying the key active compounds that positively or negatively influence specific sensory attributes (e.g., smoothness, irritability) (Pravadali-Cekic et al., 2015; Peng et al., 2022). This gap underscores the urgent need for more advanced research strategies that directly bridge sensory experience with chemical analysis.

In recent years, sensomics-based approaches have demonstrated considerable advantages in flavor analysis of complex matrices and have achieved remarkable progress in tobacco research (Carpenter et al., 2007; Kaiser et al., 2018). The core of this strategy lies in coupling instrumental separation techniques with human sensory evaluation to pinpoint key flavor components by tracking “sensory activity” (Wei et al., 2023; Wu et al., 2025). Researchers have successfully applied these methods to identify contributors to roasted-sweet and acidic notes in cigarette smoke, key sweet and sour components in cured tobacco leaves, and odor activity value profiles of tobacco with different aroma styles (e.g., fresh versus heavy aroma) (Krüsemann et al., 2017; Bernat et al., 2023).

However, existing studies on Henan tobacco have largely focused on routine chemical surveys, ecological factor influences, or macro-level sensory–chemical correlations (Zhao et al., 2013). A systematic investigation based on sensomics approaches to explore the key chemical groups governing core smoking qualities in representative Henan tobaccos remains lacking. This absence of detailed analysis limits our ability to understand the molecular origins of sensory defects and hinders the development of targeted recommendations for cultivation, harvesting, and curing practices.

To address this research gap, this study aims to establish a scientifically robust sensory quality evaluation model for tobacco leaves and apply it to assess samples from three representative production areas in Henan: Pingdingshan, Sanmenxia, and Nanyang. The research will first construct a sensory evaluation model through professional sensory assessments and multivariate statistical analysis, which will then be applied to classify 47 leaf samples based on their sensory profiles. Subsequently, sensomics techniques will be employed to identify “active fractions” responsible for key sensory differences and characterize their chemical compositions. By comparing chemical profiles of high- and low-quality samples and tracing their agronomic backgrounds, the study seeks to clarify the key aroma components affecting sensory quality and identify major agronomic influencing factors. The findings are expected to provide a theoretical foundation and technical support for precisely regulating production practices—such as fertilization and harvesting—to optimize the sensory quality of tobacco leaves.

This study is expected to establish an integrated qualitative and quantitative sensory quality evaluation system applicable to major tobacco-producing regions in Henan. Its significance lies in: deepening the understanding of the chemical basis of sensory quality formation in Henan tobaccos of different styles, particularly by elucidating the regulatory mechanisms of sensory comfort from the perspective of key active compounds; and providing direct technical and decision-making support for the “demand-driven production” and “targeted improvement” of Henan tobacco. Through precise agronomic interventions to optimize chemical composition, this research will contribute to enhancing the industrial usability and market competitiveness of Henan tobacco, thereby promoting sustainable and high-quality development of the local tobacco industry.

## 2 Materials and methodology

### 2.1 Materials, reagents and instruments

In this study, 47 tobacco leaf samples were collected from three major producing areas in Henan Province: Pingdingshan (20 samples), Nanyang (14 samples), and Sanmenxia (13 samples). These regions feature distinct geographical and climatic differences. Nanyang, located in a basin at the transition from subtropical to warm temperate zones, has a warm and humid climate (14.4-15.7°C, 700–1100 mm rainfall). In contrast, Sanmenxia is a high-altitude mountainous area with a drier and cooler temperate climate (13.7°C, 585 mm rainfall). Pingdingshan, a transitional hilly area, exhibits intermediate conditions (15°C, 726 mm rainfall). This ecological gradient provides valuable conditions for studying the impact of environmental factors on tobacco quality.

The information of tobacco leaves from different regions is shown in **Table S1**. The chemical standards utilized in this study, along with their respective purities and suppliers, were as follows: 3-hydroxy-2-butanone (97%), 2,3-butanediol (96%), ethyl palmitate (99%), and neophytadiene (90%) were obtained from Shanghai Aladdin Biochemical Technology Co., Ltd.; 1-pentanol (99%), benzyl alcohol (99%), 2,3-dihydrobenzofuran (98%), 2,6,6-trimethyl-2-cyclohexene-1,4-dione (98%), ethyl laurate (99%), farnesyl acetone (98%), 2-methoxy-4-vinylphenol (98%), and scopoletin (98%) were acquired from Beijing Innochem Science & Technology Co., Ltd.; 2,3-hexanedione (99%) and 2,5-diethyl-3,6-dimethylpyrazine (95%) were provided by Shanghai Titan Scientific Co., Ltd.; 6-methyl-2-heptanol (99%), linalool (95%), and damascone (90%) were purchased from Sigma-Aldrich (USA); benzaldehyde (99%) and *p*-tolualdehyde (98%) were sourced from Alfa Aesar (USA); 6-methyl-5-hepten-2-ol (98%), phenylacetaldehyde (95%), and dihydroactinidiolide (97%) were supplied by Shanghai Macklin Biochemical Co., Ltd.; phenethyl alcohol (99%) was obtained from Beijing J&K Scientific Ltd.; 2,3-dihydro-3,5-dihydroxy-6-methyl-4(H)-pyran-4-one (95%) was provided by Chemicell GmbH; α-Ionone (90%) and ethyl myristate (98%) were purchased from Tokyo Chemical Industry Co., Ltd. (TCI); megastigmatrienone (50%) was acquired from Shanghai ZhiPu Industrial Co., Ltd.; α-allyl ionone (85%) was supplied by Shanghai Topfine Chemical Co., Ltd.; nicotine (95%) was obtained from Toronto Research Chemicals (TRC, Canada); styrallyl propionate (98%) was purchased from Acros Organics (USA).

The analysis was performed using an Agilent 8890-5977B gas chromatography–mass spectrometry (GC–MS) system. Sample weighing was conducted on an AL204 electronic analytical balance (sensitivity: 0.0001 g), temperature-controlled steps were carried out using a HH-S8A constant-temperature water bath, and sample preparation involved the use of 0.22 μm ultrafiltration membranes and 5 mL disposable sterile syringes.

### 2.2 Sample pre-treatment

For the analysis of aroma components, 2.5 g of tobacco leaf powder was accurately weighed into a 50 mL glass-stoppered conical flask. Then, 20 mL of an ethanol solution containing an internal standard (1-Phenylethyl propionate, 0.8608 μg mL^-1^) was added. The mixture was heated in a water bath at 80°C for 2 hours. After cooling and settling, the supernatant was collected and passed through a 0.22 μm ultrafiltration membrane. The initial 10 drops of the filtrate were discarded, and the subsequent filtrate was subjected to GC-MS analysis. Each sample was analyzed in duplicate.

### 2.3 Instrumental analysis

A DB-5MS capillary column (60 m × 250 μm × 0.25 μm) was used for the separation of aroma components. Helium was employed as the carrier gas at a constant flow rate of 1.0 mL min^-1^. The injector temperature was maintained at 250°C, and samples were introduced via a direct, splitless injection mode. The oven temperature program was set as follows: initial temperature 50°C, increased at a rate of 3 °C/min to a final temperature of 260°C. The GC-MS transfer line temperature was set at 270°C. Electron impact (EI) ionization was applied at 70 eV, with the ion source and quadrupole temperatures held at 230°C and 150°C, respectively. Data acquisition was performed in selected ion monitoring (SIM) mode.

For the analysis of nicotine, a DB-WAXETR capillary column (60 m × 0.25 mm × 0.25 μm) was utilized. The oven temperature program was modified as follows: initial temperature 100°C, ramped at 6°C min^-1^ to 220°C. All other instrumental parameters remained consistent with those described above.

Differential components identified in tobacco from Western and Central Henan regions were confirmed using commercially available reference standards. This process enabled the precise identification of the key chemical groups responsible for the sensory quality of Henan tobacco leaves and facilitated the establishment of a reliable quantitative method. Detailed parameters, including retention times and qualitative/quantitative ions for all target analyses, are provided in **Table 1**. In the quantitative analysis of nicotine, the retention times, quantitative ions and qualitative ions of nicotine and the internal standard are listed in **Supplementary Table 2**. The specific selection criteria were as follows: refer to **SI**.

**Table 1:** The retention times, quantitative ions and qualitative ions of 29 analyte compounds.

No.	Compound	RT/min	Quantitative ion (m/z)	Qualitative ion (m/z)	
1	3-Hydroxy-2-butanone	7.385	45	43	88
2	1-Pentanol	8.46	55	57	70
3	2,3-Butanediol	8.78	45	57	75
4	2,3-Hexanedione	8.894	114	43	71
5	6-Methyl-2-heptanol	15.231	84	69	97
6	Benzaldehyde	15.356	106	77	105
7	6-Methyl-5-hepten-2-ol	16.477	110	95	128
8	Benzyl alcohol	18.486	108	79	107
9	Phenylacetaldehyde	19.038	91	92	120
10	Coumaran	20.755	120	91	119
11	*p*-Tolualdehyde	20.995	119	91	120
12	Linalool	21.547	93	121	136
13	Phenethyl alcohol	22.268	91	92	122
14	2,3-Dihydro-3,5-dihydroxy-6-methyl-4(H)-pyran-4-one	23.771	144	101	115
15	2,6,6-Trimethyl-2-cyclohexene-1,4-dione	23.824	96	137	152
16	2,5-Diethyl-3,6-dimethylpyrazine	27.485	149	163	164
17	2-Methoxy-4-vinylphenol	31.601	150	107	135
18	Damascenone	35.981	177	123	192
19	α-Ionone	36.54	121	136	192
20	Dihydroactinidiolide	41.221	111	137	180
21	Megastigmatrienone I	42.236	190	148	175
22	Megastigmatrienone II	42.987	190	148	175
23	Ethyl laurate	43.362	88	101	157
24	Megastigmatrienone III	44.299	190	148	175
25	Megastigmatrienone IV	44.81	190	148	175
26	Allyl ionone	47.078	232	135	161
27	Ethyl tetradecanoate	50.772	101	157	213
28	Neophytadiene	52.319	123	109	137
29	Farnesyl acetone	54.874	136	107	125
30	Scopoletin	57.704	192	149	177
31	Ethyl palmitate	57.501	101	157	241
32	Phenylethyl propionate (internal standard)	30.018	122	104	178

### 2.4 Sensory evaluation methods

Sensory evaluation followed the national industry standard YC/T 530: Flue−cured Tobacco - Sensory Evaluation Method for Leaf Quality and Style, with an additional industrial usability attribute included to assess the feasibility of using the leaves in cigarette formulations. Seven professional evaluators, each holding a certified cigarette sensory inhalation qualification, formed the panel. Prior to evaluation, all samples were conditioned under standardized environmental conditions (temperature: 22 ± 2°C; relative humidity: 60 ± 5%). Samples were coded with random three-digit numbers and presented to panelists in a randomized and blinded order to minimize bias. Each sample was evaluated in triplicate across independent sessions. Evaluated attributes included aroma/style features (e.g., floral/fruit, green/woody, roasted/sweet), aroma intensity/volume, irritation, aftertaste, off−odor, ash color (gray, defined according to YC/T 530 as the appearance of ash after combustion), and industrial usability; quality attributes and industrial usability were scored on a 0–9 scale while aroma/style attributes were scored on a 0–5 scale. Each sample was independently evaluated by all seven panelists and the final attribute score for each sample was calculated as the arithmetic mean of the seven evaluators’ ratings.

## 3 Results and discussion

### 3.1 Methodological examination and quantitative analysis

This study conducted a qualitative analysis of the key components related to the sensory quality of tobacco leaves from the western and eastern regions of Henan Province. The volatile compounds in tobacco leaf samples from these two regions were compared and analyzed using gas chromatography–mass spectrometry (GC-MS), and their compositional differences were systematically examined. The volatile components were identified by searching the NIST19 mass spectrometry database, with a match threshold greater than 85% used for compound verification, supplemented by confirmation with standard substances. Ultimately, a total of 28 characteristic components exhibiting regional differences were identified (**Table 2**). These components provide important insights into the material basis underlying the differences in sensory quality between tobacco leaves from the two regions.

**Table 2:** The linear range, standard curve, correlation coefficient and relative standard deviation of the quantitative methods for 28 components and nicotine.

Compound	Linearity range (μg mL^-1^)	Standard curve	R^2^	LOD (ng mL^-1^)	LOQ (ng mL^-1^)	RSD/%
3-Hydroxy-2-butanone	0.1700~3.4000	y=0.7273x-0.00763	0.9999	57.92	193.07	7.83
1-Pentanol	0.0480~0.960	y=0.4191x-0.00106	0.9997	1.79	5.96	5.64
2,3-Butanediol	1.2460~24.920	y=0.9765x-0.4924	0.9997	45.48	151.60	2.62
2,3-Hexanedione	0.0044~0.0880	y=0.03916x+0.000052	0.9998	0.97	3.25	9.59
6-Methyl-2-heptanol	0.00524~0.1048	y=0.1513x+0.00029	0.9997	0.37	1.23	6.98
Benzaldehyde	0.0488~0.9760	y=1.013x-0.02749	0.9998	1.84	6.13	2.22
6-Methyl-5-hepten-2-ol	0.00492~0.0984	y=0.2271x+0.00011	0.9998	0.55	1.82	4.59
Benzyl alcohol	0.4496~8.992	y=0.5759x-0.3732	0.9983	0.28	0.92	0.02
Phenylacetaldehyde	0.6056~12.112	y=1.531x-0.6764	0.9991	4.76	15.86	0.39
Coumaran	0.0480~0.960	y=1.499x-0.01609	0.9998	0.61	2.04	1.28
*p*-Tolualdehyde	0.00492~0.0984	y=1.018x-0.004949	0.9998	0.33	1.09	1.50
Linalool	0.0872~1.7440	y=0.5329x-0.01759	0.9999	0.60	2.00	0.54
Phenethyl alcohol	0.4996~9.9920	y=0.8910x-0.6552	0.9963	4.48	14.94	0.24
2,3-Dihydro-3,5-dihydroxy-6-methyl-4(H)-pyran-4-one	0.0436~0.8720	y=0.01134x-0.00037	0.9996	3.35	11.17	3.98
2,6,6-Trimethyl-2-cyclohexene-1,4-dione	0.03304~0.6608	y=0.9341x-0.01194	0.9998	0.70	2.33	1.61
2,5-Diethyl-3,6-dimethylpyrazine	0.00464~0.0928	y=1.747x-0.000417	0.9998	0.08	0.28	3.01
2-Methoxy-4-vinylphenol	1.3144~26.2880	y=0.06891x-0.0234	0.9996	1.84	6.12	0.22
Damascenone	0.0516~1.0320	y=1.140x-0.03916	0.9994	0.64	2.12	0.65
α-Ionone	0.0432~0.864	y=1.026x-0.03465	0.9994	0.44	1.46	0.46
Dihydroactinidiolide	0.2444~4.888	y=0.8182x-0.3308	0.9946	1.26	4.21	0.13
Megastigmatrienone	6.9500~139.00	y=2.2317x+8.7308	0.9992	19.59	65.30	0.09
Ethyl laurate	0.00668~0.1336	y=1.173x-0.01700	0.9924	0.02	0.06	0.05
Allyl ionone	0.005066~0.10132	y=0.2118x-0.00086	0.9999	0.09	0.29	0.86
Ethyl tetradecanoate	0.00516~0.1032	y=0.9375x-0.00474	0.9996	0.32	1.08	2.54
Neophytadiene	3.6800~73.600	y=0.3280x+0.8768	0.9902	14.25	47.50	0.24
Farnesyl acetone	0.5160~10.320	y=0.5141x-0.1859	0.9999	2.15	7.16	0.24
Scopoletin	2.2000~44.000	y=0.4625x-1.109	0.9999	0.17	0.58	0.00
Ethyl palmitate	0.1588~3.1760	y=1.189x-0.0777	0.9992	9.46	31.55	4.85
Nicotine	0.5680~11.3600	y=1.3143x+0.0316	0.9995	0.20	0.67	9.14

The linear range, standard curve, correlation coefficient, etc. of the quantitative methods for 28 components and nicotine were shown in **Table 2**. The correlation coefficients were all higher than 0.99, and the RSDs are all within 10%, indicating that the precision of this method meets the requirements and can be used for quantitative analysis.

**Figure 1:**
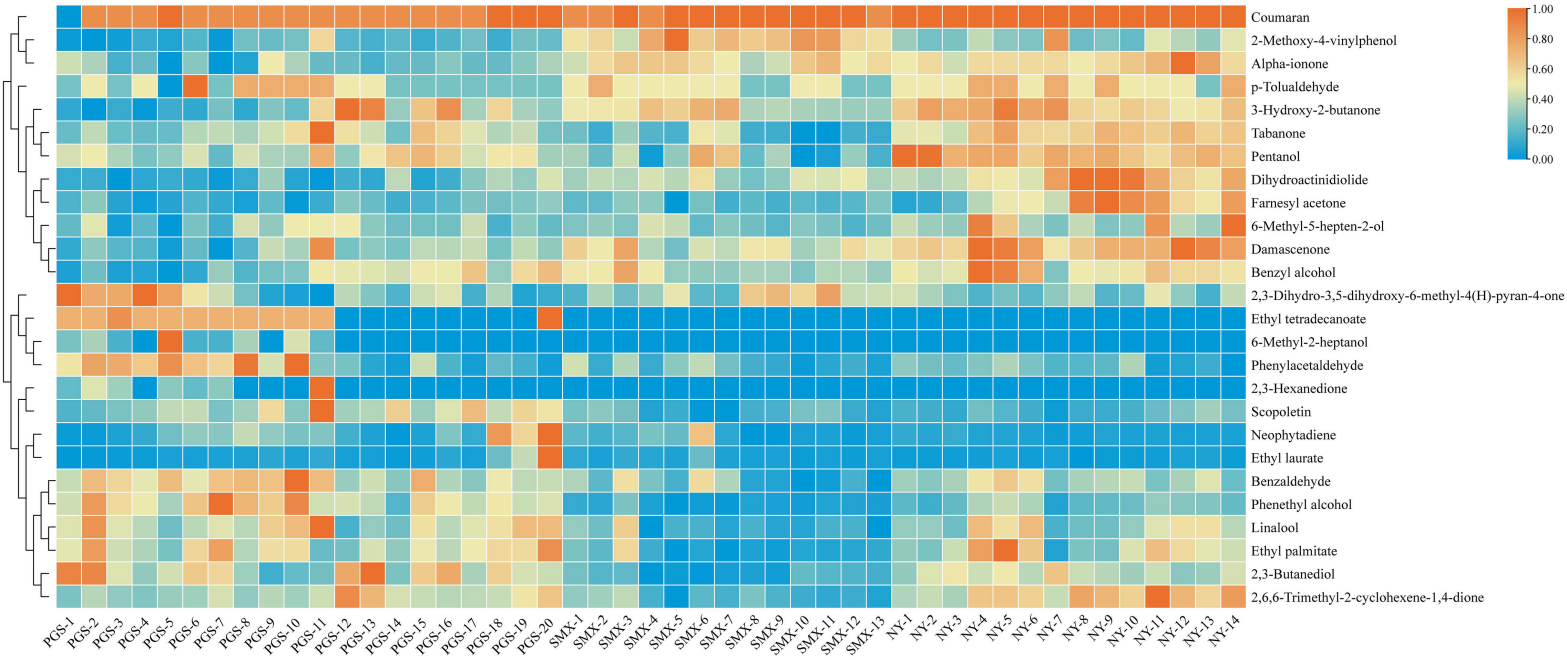
Heat map of the content distribution of 28 components in 47 tobacco samples.

A previously established quantitative method was employed to determine the concentrations of 28 chemical constituents across 47 tobacco leaf samples (Lin et al., 2025; Yuan et al., 2025). The content distribution of these compounds is visually represented in the heatmap shown in **Figure 1**. Heatmap analysis revealed that benzyl alcohol, damascone, 6-methyl-5-hepten-2-ol, dihydroactinidiolide, farnesyl acetone, 2,3-dihydrobenzofuran, 2-methoxy-4-vinylphenol, 3-hydroxy-2-butanone, α-ionone, *p*-tolualdehyde, megastigmatrienone, and neophytadiene (corresponding to labels A12, A11, A9, A19, A18, A25, A21, A7, A17, A10, A6, and A3, respectively) exhibited relatively higher levels in samples from the Nanyang region. In contrast, tobacco leaves from the Pingdingshan region were characterized by elevated concentrations of 2,3-butanediol, 2,6,6-trimethyl-2-cyclohexene-1,4-dione, benzaldehyde, phenethyl alcohol, linalool, and 1-pentanol (A5, A1, A26, A20, A14, and A13, respectively).

Based on the heatmap patterns, it was inferred that the Nanyang samples, with their higher abundances of benzyl alcohol, β-damascone, and megastigmatrienone—degradation products often associated with carotenoid metabolism—typically impart floral, fruity, and sweet aromatic notes, suggesting a potentially more pronounced aromatic quality (Jiang et al., 2024). Conversely, the Pingdingshan samples were enriched with alcoholic constituents such as 2,3-butanediol, phenethyl alcohol, and linalool. While these compounds also contribute to the overall tobacco aroma, their sensory character is generally lighter and more delicate; however, some, like 1-pentanol, may introduce harsh notes at elevated concentrations. This regional divergence in chemical profile is likely attributable to agronomic and environmental factors specific to Pingdingshan—such as climate, soil properties, or cultivation practices—which may favor metabolic pathways leading to the accumulation of these alcohols.

Detailed quantitative results are provided in **Supplementary Table 3**. Descriptive statistics, including maximum, minimum, mean, and standard deviation for all components, are summarized in **Supplementary Table 4**. Terpenoids and phenylpropanoid derivatives were identified as the predominant high-abundance compounds, forming the foundational aroma base of the tobacco leaves (Chen et al., 2025; Wei et al., 2025). Among all quantified constituents, neophytadiene recorded the highest mean concentration (970.34 µg g^-1^), with a standard deviation of 161.16 and a coefficient of variation (CV) of 16.61%, indicating relatively stable distribution across samples and confirming its role as the major terpenoid. This was followed by 2-methoxy-4-vinylphenol (mean: 424.55 µg g^-1^) and scopoletin (420.18 µg g^-1^), both of which showed considerable sample-to-sample variation (CV = 43.37% and 47.67%, respectively), suggesting that their levels are susceptible to environmental or cultivation-related influences. Megastigmatrienone was present at a substantial mean level of 183.56 µg g^-1^ and displayed relatively low variability (CV = 12.65%), along with a high detection frequency, marking it as a consistent contributor to the leaf’s aroma profile.

Medium-abundance compounds, including 2,3-Dihydro-3,5-dihydroxy-6-methyl-4(H)-pyran-4-one (34.76 µg g^-1^), 2,3-butanediol (20.56 µg g^-1^), phenethyl alcohol (20.59 µg g^-1^), and phenylacetaldehyde (18.75 µg g^-1^), primarily originate from the degradation and transformation of carbohydrates, amino acids, and aromatic precursors (Okal et al., 2025). These components are critical in the development of sweet, fruity, and roasted notes during curing and storage stages. Low-abundance compounds, such as 1-pentanol and linalool, exhibited mean concentrations mostly below 1 µg g^-1^ and high CV values, reflecting significant inter-sample fluctuations. Despite their low levels, these compounds play a discernible role in aroma modulation, enhancing the complexity, layering, and overall harmony of the scent profile.

In summary, the chemical composition of the tobacco leaves exhibited a characteristic pattern defined by a limited number of dominant high-abundance compounds coexisting with a diverse array of low-abundance aroma-modifying substances. This structure implies that while the overall aromatic quality is primarily determined by key constituents, it is further fine-tuned through the synergistic influence of numerous minor components, ultimately giving rise to the complex and regionally distinct chemical signatures observed.

### 3.2 Correlation analysis based on characteristic components

The chemical–sensory relationships identified in this study are highly consistent with previously reported findings on the aroma−forming mechanisms of flue−cured tobacco. Prior studies by Fan et al (Fan et al., 2026). demonstrated that carotenoid−derived volatiles such as β−damascone, megastigmatrienone, and dihydroactinidiolide are major contributors to sweet, floral, and fruity sensory attributes, which aligns closely with the positive correlations observed in our dataset. Similarly, the roles of phenylpropanoid−derived compounds—including benzaldehyde and phenethyl alcohol—in enhancing aroma richness and smoothness have been repeatedly confirmed in earlier work (Weber Böhlen et al., 2026), supporting the associations identified in our correlation analysis. Furthermore, the elevated levels of alcohols and nitrogen−related volatiles in regions with higher nitrogen availability are consistent with reports by Ren et al (Ren et al., 2025). describing the influence of nitrogen metabolism on irritation and smoke purity. These consistencies collectively reinforce the robustness of our sensory–chemical evaluation model and demonstrate that the key aroma contributors identified in Henan tobacco follow well−established biochemical patterns documented in the literature.

**Figure 2:**
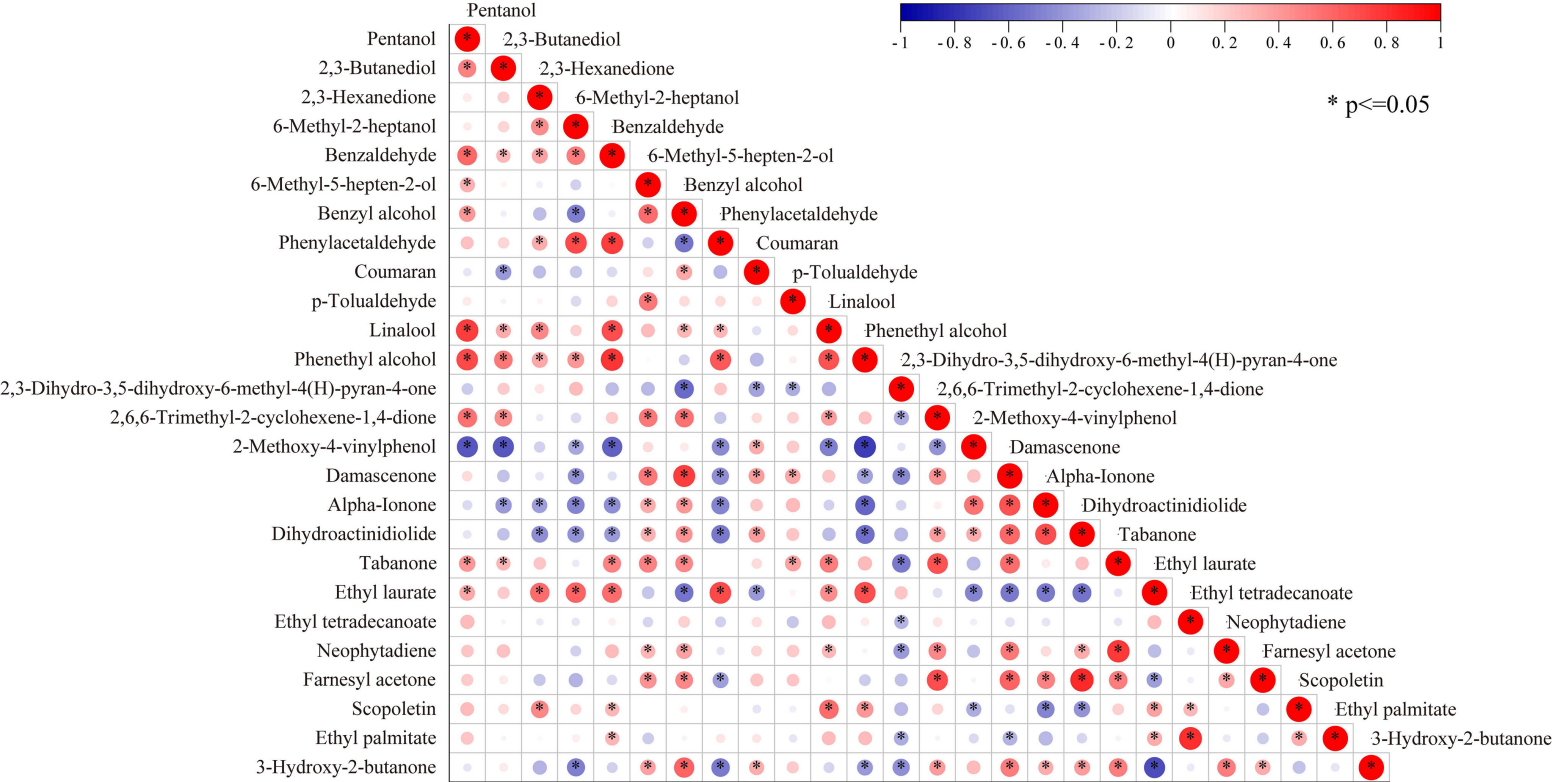
Correlation analysis of aroma components.

A comprehensive Pearson correlation analysis was systematically performed to evaluate the interrelationships among the identified aroma components in the tobacco leaf samples. The resultant correlation matrix, visually summarized in **Figure 2**, provided significant insights into the complex interaction patterns between different chemical classes. Statistically significant correlations (*p* < 0.05) were predominantly observed between compounds belonging to alcoholic and aldehydic classes. Specifically, linalool and phenethyl alcohol demonstrated strong positive correlations with 1-pentanol and benzaldehyde. Similarly, a markedly strong positive correlation was identified between p-tolualdehyde and 6-methyl-5-hepten-2-ol. In contrast, the phenolic compound 2-methoxy-4-vinylphenol displayed significant negative correlations (*p* < 0.05) with a range of compounds, including 1-pentanol, 2,3-butanediol, benzaldehyde, phenylacetaldehyde, linalool, and phenethyl alcohol. Concurrently, analysis of the ester compound ethyl laurate revealed its strong positive correlations with 2,3-hexanedione, 6-methyl-2-heptanol, benzaldehyde, phenylacetaldehyde, and phenethyl alcohol, whereas it exhibited a strong negative correlation with 3-hydroxy-2-butanone (*p* < 0.05). Of particular note, 2,5-diethyl-3,6-dimethylpyrazine and α-allyl ionone were found to have no statistically significant correlations with any other analyzed compounds at the 0.05 significance level. Consequently, in accordance with this statistical finding, these two components were rationally excluded from all subsequent analytical procedures to focus the investigation on the more interactive and potentially impactful aroma compounds.

### 3.3 Multivariate statistical analysis

**Figure 3:**
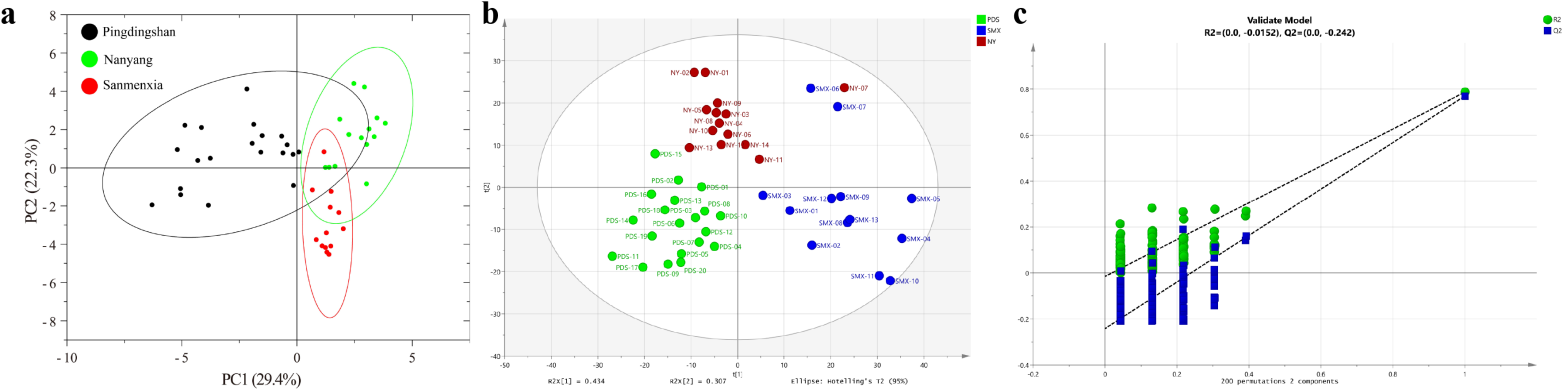
**(a)** Principal component analysis (PCA) of 26 components. **(b)** Partial Least Squares Discriminant Analysis (PLS-DA) based on 26 components and **(c)** Construction of the prediction model.

Principal component analysis (PCA) was performed to elucidate the overall distribution characteristics of the samples based on 26 compounds. As shown in **Figure 3a**, the cumulative contribution rate of the first two principal components reached 52%, with PC1 accounting for 29.4% and PC2 for 22.3%, indicating that a substantial portion of the structural information in the data could be captured by these components. Meanwhile, volatile constituents with significant contributions to each principal component were identified. Representative compounds in PC1 included 3-hydroxy-2-butanone (creamy, sweet aroma), 6-methyl-2-heptanol (fruity, green note), benzyl alcohol (subtle floral scent), damascone (intense fruity and tea-like aroma), α-ionone (violet-like floral and woody note), dihydroactinidiolide (peach-like fruity and tea aroma), and farnesyl acetone (fresh sweet floral and balsamic note). These components predominantly exhibit sweet, fruity, and floral characteristics, commonly found in tobacco, and contribute significantly to the “sweetness” and “fruity nuance” in tobacco quality. The main representative compounds in PC2 were 1-pentanol (wine-like, fruity aroma), linalool (fresh floral and woody note), and megastigmatrienone (hay-like, typical tobacco aroma). These constituents collectively presented green, woody, and wine-like tones, reflecting the refreshing and vegetal aspects of tobacco, potentially associated with raw material traits or volatile profiles formed during certain fermentation processes. In summary, PCA not only revealed the distribution structure of the samples in the reduced-dimensional space but also reflected potential differences in aroma composition among tobacco samples through key compound information, providing a chemical basis for understanding their flavor characteristics.

To further validate the discriminatory power of the identified chemical constituents and to assess the predictive ability of the model in a supervised framework, Partial Least Squares Discriminant Analysis (PLS-DA) was performed using the 26 quantified compounds as the X−matrix and the three growing regions (Pingdingshan, Sanmenxia, Nanyang) as the categorical Y−response (**Figures 4b, c**). The analysis was carried out using SIMCA 14.1 (Umetrics, Sweden) with unit variance (UV) scaling and seven−fold cross−validation. Model performance was evaluated by the cumulative explained variation in the X matrix (R²X), the cumulative explained variation in the Y matrix (R²Y), and the cumulative predicted variation (Q²). To exclude overfitting, a permutation test (200 permutations) was conducted. The achieved R²Y = 0.71 and Q² = 0.70, indicating good fit and satisfactory predictive capability. The permutation test showed that all Q² values of the permuted models were lower than the original Q² and the regression line of Q² intersected the Y−axis below zero, confirming the absence of overfitting.

Variable Importance in Projection (VIP) scores were used to identify compounds most influential for regional discrimination. As shown in **Supplementary Table 5**, four compounds exhibited VIP values greater than 1, namely 2-methoxy-4-vinylphenol (VIP = 3.0), neophytadiene (VIP = 2.9), scopoletin (VIP = 2.6), and megastigmatrienone (VIP = 1.0). These compounds therefore represent the core chemical signatures differentiating the three Henan tobacco regions. Notably, 2-methoxy-4-vinylphenol, neophytadiene, and megastigmatrienone are also among the 13 key aroma components identified through correlation analysis (Section 3.4), reinforcing their central role in determining sensory quality. The emergence of scopoletin-a coumarin derivative with known biological activities-as a high−VIP compound suggests that it may also contribute to regional sensory differences, warranting further investigation. The PLS-DA results thus provide a supervised statistical confirmation of the key chemical drivers of tobacco sensory quality and demonstrate the model’s potential for predicting the regional origin and, by extension, the sensory characteristics of unknown samples based on their chemical profiles.

PCA and PLS-DA serve complementary roles in our analytical framework. PCA, as an unsupervised exploratory technique, reveals the inherent structure and natural groupings within the dataset without prior knowledge of sample classifications, providing an unbiased overview of sample distribution and identifying major sources of variation through key contributing compounds. In contrast, PLS-DA operates as a supervised method that leverages known class information (the three growing regions) to maximize separation between predefined groups and identify the most discriminative variables through VIP scoring. This complementary approach ensures that our findings are both data-driven (through PCA’s exploratory nature) and hypothesis-validated (through PLS-DA’s targeted discrimination), creating a robust analytical pipeline where PCA identifies potential patterns and PLS-DA confirms and refines these patterns with respect to regional origin, thereby strengthening the reliability of our chemical marker identification for tobacco sensory quality assessment.

### 3.4 Sensory evaluation analysis

Pearson correlation analysis was conducted to investigate the relationships between aroma components and sensory attributes, with the results summarized in **Table 3**. Multiple aroma components exhibited highly significant or significant positive correlations with aroma quality, off-odor, industrial usability, and hay-like aroma. These included 3-hydroxy-2-butanone, benzaldehyde, p-tolualdehyde, linalool, 2-methoxy-4-vinylphenol, α-ionone, 2,3-dihydrobenzofuran, megastigmatrienone, and neophytadiene.

**Table 3:** Correlation between important aroma components and sensory indicators.

Compound	Quality of aroma	Volume of aroma	Concentration	Strength	Offensive taste	Irritancy	After taste	Gray	Commercial availability	Hay incense	burnt-sweetness aroma	Mellow-sweet aroma	burnt aroma
3-Hydroxy-2-butanone	0.423^**^	0.281	-0.014	-0.413^**^	0.339^*^	0.206	0.254	0.610^**^	0.419^**^	0.406^*^	0.358^*^	0.301	-0.309
1-Pentanol	-0.192	-0.081	0.204	0.210	-0.139	-0.022	-0.146	-0.460^**^	-0.196	-0.297	-0.101	-0.204	0.328^*^
2,3-Butanediol	0.162	0.113	-0.089	-0.446^**^	0.274	0.154	0.150	-0.007	0.157	0.216	0.399^*^	0.275	-0.335^*^
2,3-Hexanedione	-0.103	0.013	0.030	0.374^*^	-0.007	0.070	0.011	-0.220	-0.192	-0.173	-0.324^*^	-0.051	0.091
6-Methyl-2-heptanol	0.071	0.128	-0.026	0.202	0.167	0.186	0.140	-0.205	0.061	0.076	-0.118	-0.013	-0.132
Benzaldehyde	0.371^*^	0.282	-0.073	-0.163	0.358^*^	0.260	0.332^*^	0.550^**^	0.318^*^	0.380^*^	0.154	0.287	-0.237
6-Methyl-5-hepten-2-ol	0.449^**^	0.366^*^	-0.007	-0.079	0.287	0.181	0.203	0.633^**^	0.390^*^	0.144	0.157	0.194	-0.062
Benzyl alcohol	0.376^*^	0.239	0.093	-0.229	0.220	0.204	0.169	0.570^**^	0.381^*^	0.259	0.262	0.177	-0.100
Phenylacetaldehyde	0.293	0.274	-0.165	-0.009	0.374^*^	0.375^*^	0.345^*^	0.325^*^	0.246	0.273	-0.041	0.246	-0.166
Coumaran	0.386^*^	0.274	-0.146	-0.301	0.246	0.184	0.258	0.632^**^	0.379^*^	0.396^*^	0.234	0.291	-0.197
p-Tolualdehyde	0.472^**^	0.349^*^	-0.149	-0.290	0.406^*^	0.301	0.338^*^	0.702^**^	0.425^**^	0.389^*^	0.233	0.345^*^	-0.282
Linalool	0.381^*^	0.253	-0.123	-0.240	0.352^*^	0.257	0.311	0.564^**^	0.340^*^	0.337^*^	0.213	0.291	-0.246
Phenethyl alcohol	-0.067	0.060	0.049	0.086	0.046	0.080	0.115	-0.250	-0.110	-0.062	0.007	0.098	0.037
2,3-Dihydro-3,5-dihydroxy-6-methyl-4(H)-pyran-4-one	0.099	0.076	-0.283	-0.224	0.206	0.178	0.124	0.028	0.075	0.106	-0.012	0.206	-0.203
2,6,6-Trimethyl-2-cyclohexene-1,4-dione	0.150	0.064	-0.124	-0.385^*^	0.114	-0.038	0.025	0.304	0.128	0.113	0.227	0.052	-0.079
2-Methoxy-4-vinylphenol	0.438^**^	0.284	-0.128	-0.179	0.345^*^	0.271	0.295	0.642^**^	0.393^*^	0.342^*^	0.182	0.308	-0.245
Damascenone	0.425^**^	0.254	-0.127	-0.236	0.332^*^	0.213	0.229	0.671^**^	0.374^*^	0.283	0.122	0.164	-0.130
α-Ionone	0.430^**^	0.266	-0.176	-0.310	0.394^*^	0.271	0.315	0.642^**^	0.387^*^	0.372^*^	0.204	0.281	-0.275
Dihydroactinidiolide	0.322^*^	0.156	-0.269	-0.382^*^	0.303	0.189	0.214	0.601^**^	0.283	0.261	0.163	0.191	-0.201
Megastigmatrienone	0.417^**^	0.270	-0.127	-0.306	0.367^*^	0.213	0.293	0.647^**^	0.365^*^	0.353^*^	0.228	0.263	-0.254
Ethyl laurate	-0.168	-0.012	-0.094	0.295	-0.032	0.042	0.040	-0.330^*^	-0.215	-0.179	-0.235	-0.026	0.072
Ethyl tetradecanoate	-0.001	-0.022	0.064	-0.002	0.001	0.009	0.081	0.111	0.055	0.062	0.261	0.147	-0.104
Neophytadiene	0.409^**^	0.275	-0.122	-0.362^*^	0.332^*^	0.198	0.289	0.629^**^	0.404^*^	0.385^*^	0.268	0.266	-0.261
Farnesyl acetone	0.124	-0.033	-0.357^*^	-0.339^*^	0.198	0.014	0.033	0.410^**^	0.043	0.007	0.008	-0.050	-0.011
Ethyl palmitate	0.154	0.093	0.077	-0.049	0.156	0.107	0.201	0.397^*^	0.169	0.197	0.268	0.301	-0.189

Note: “*” indicates significant correlation; “**” indicates extremely significant correlation.

Aroma quality was also highly significantly positively correlated with 6-methyl-5-hepten-2-ol and β-damascone, and significantly positively correlated with benzyl alcohol, linalool, and dihydroactinidiolide. Industrial usability showed additional significant positive correlations with 6-methyl-5-hepten-2-ol, benzyl alcohol, and damascone. Off-odor was further highly significantly positively correlated with 6-methyl-5-hepten-2-ol, benzyl alcohol, damascone, and dihydroactinidiolide, while ethyl palmitate and phenylacetaldehyde showed significant positive correlations with this attribute. In addition, 3-hydroxy-2-butanone and 2,3-butanediol were significantly positively correlated with burnt-sweet aroma. Notably, 3-hydroxy-2-butanone, benzaldehyde, p-tolualdehyde, linalool, 2-methoxy-4-vinylphenol, α-ionone, damascone, megastigmatrienone, and neophytadiene showed significant positive correlations with off-odor intensity.

Based on these correlation results, 13 components—namely 3-hydroxy-2-butanone, benzaldehyde, p-tolualdehyde, linalool, 2-methoxy-4-vinylphenol, α-ionone, 2,3-dihydrobenzofuran, megastigmatrienone, neophytadiene, 6-methyl-5-hepten-2-ol, benzyl alcohol, damascone, and dihydroactinidiolide—were identified as key components strongly associated with the sensory quality of tobacco leaves and should be prioritized in future tobacco quality evaluation studies.

The agronomic associations observed between cultivation factors and key aroma compounds can be plausibly interpreted through crop physiological and metabolic mechanisms. Nitrogen fertilization, for example, alters the plant carbon–nitrogen balance and amino−acid metabolism (Duan et al., 2024), which in turn can modulate the synthesis and accumulation of volatile compounds derived from amino acids and carbohydrates (such as certain alcohols, aldehydes, ketones and pyrazines); thus, correlations between fertilization intensity or timing and elevated levels of alcohols and phenyl−derived volatiles are consistent with a nitrogen−driven shift in precursor availability and downstream volatile formation. Single−leaf weight is closely linked to plant vigor, leaf thickness and dry−matter accumulation; larger, denser leaves typically exhibit different thermal and oxidative behavior during curing and combustion, which can favor the formation or retention of carotenoid−derived degradation products (e.g., β−damascone, megastigmatrienone) and other terpenoid signatures. Harvest method and timing (manual versus mechanical harvest, and early versus late harvest) influence the degree of mechanical injury and the maturity state of the tissue, thereby affecting enzyme−mediated oxidation and microbial transformations that produce or deplete labile aroma precursors; mechanical damage, for instance, can accelerate lipid oxidation and the release of aldehydes and ketones that alter irritation or off−odor profiles (Lin et al., 2021). Finally, plant growth vigor as shaped by stand density, light interception and ventilation affects photosynthate partitioning and secondary metabolism, with downstream effects on terpenoid, phenolic and carotenoid pathways that collectively shape regional aroma styles.

The distribution patterns of the 13 key aroma components across 47 tobacco leaf samples were statistically analyzed in relation to four agronomic factors: fertilization, plant growth vigor, harvesting method, and single-leaf weight. The results are summarized in **Table 4**.

**Table 4:** The average values, standard deviations, and coefficient of variation of the contents of 13 key aroma components under different treatment methods.

Compound	Fertilization			Growth vigour			Harvesting method			Leaf weight		
	Average (×10^-3^)	Standard deviation (×10^-3^)	Coefficient of variation/%	Average (×10^-3^)	Standard deviation (×10^-3^)	Coefficient of variation/%	Average (×10^-3^)	Standard deviation (×10^-3^)	Coefficient of variation/%	Average (×10^-3^)	Standard deviation (×10^-3^)	Coefficient of variation/%
3-Hydroxy-2-butanone	0.20	0.19	98.19	0.18	0.07	40.40	0.20	0.05	23.28	0.24	0.08	31.62
Benzaldehyde	0.06	0.02	35.67	0.04	0.00	11.55	0.04	0.01	13.69	0.04	0.01	16.13
6-Methyl-5-hepten-2-ol	0.07	0.04	55.15	0.05	0.01	17.83	0.06	0.02	24.35	0.07	0.02	29.68
Benzyl alcohol	0.86	0.52	60.43	0.89	0.20	22.95	1.10	0.15	13.62	1.10	0.14	12.97
Coumaran	0.01	0.00	58.27	0.00	0.00	12.64	0.01	0.00	16.23	0.00	0.00	21.06
p-Tolualdehyde	0.00	0.00	44.33	0.00	0.00	11.30	0.00	0.00	20.33	0.00	0.00	24.24
Linalool	0.02	0.01	36.26	0.02	0.00	9.51	0.02	0.00	12.58	0.02	0.00	16.75
2-Methoxy-4-vinylphenol	36.04	38.70	107.37	17.81	3.35	18.81	47.41	14.48	30.55	27.48	15.79	57.47
Damascenone	0.07	0.04	53.86	0.06	0.01	9.59	0.08	0.01	8.49	0.08	0.01	14.00
α-Ionone	0.02	0.01	51.78	0.02	0.00	11.53	0.02	0.00	18.59	0.02	0.00	20.41
Dihydroactinidiolide	0.51	0.28	55.14	0.36	0.07	19.28	0.53	0.10	19.31	0.53	0.14	27.47
Megastigmatrienone	12.36	5.82	47.13	9.63	1.04	10.85	9.85	1.10	11.19	11.59	2.31	19.93
Neophytadiene	66.85	36.03	53.90	48.70	7.30	14.98	48.46	3.64	7.51	65.19	16.25	24.93

Overall, fertilization exhibited the most substantial influence on the variation of aroma components (Yan et al., 2020; Liang et al., 2026), with coefficients of variation (CV) ranging from 35.67% to 107.37% across the 13 compounds. Notably, 3-hydroxy-2-butanone and 2-methoxy-4-vinylphenol were the most sensitive to fertilization changes. In contrast, plant growth vigor had a relatively minor impact, with only 3-hydroxy-2-butanone and benzyl alcohol showing CVs exceeding 20% (40.40% and 22.95%, respectively), while the remaining components varied by less than 20%. Under different harvesting methods, 2-methoxy-4-vinylphenol was the most affected, with a CV exceeding 30%. Variations in single-leaf weight primarily influenced 2-methoxy-4-vinylphenol, followed by 3-hydroxy-2-butanone.

Furthermore, linalool was largely unaffected by plant growth vigor and only minimally influenced by harvesting method and single-leaf weight, with fertilization being its dominant influencing factor. Similarly, damascone showed negligible sensitivity to plant growth vigor and harvesting method, was slightly affected by single-leaf weight, and was most strongly influenced by fertilization. Neophytadiene was almost unaffected by harvesting method, minimally influenced by plant growth vigor, and predominantly modulated by fertilization.

These findings demonstrate that targeted adjustments in cultivation or harvesting practices can effectively modulate the composition of key aroma components, thereby enabling deliberate quality control of tobacco leaves.

### 3.5 Biosynthetic pathways and metabolic regulation of key aroma components

The 13 key aroma components identified in this study originate from diverse biosynthetic pathways, and their accumulation is subject to complex metabolic regulation influenced by genetic, environmental, and agronomic factors. Understanding these pathways provides a mechanistic basis for interpreting the observed correlations between sensory attributes and chemical composition, and for designing targeted agronomic interventions.

Among these compounds, several are derived from the degradation of carotenoids, which are tetraterpenoids synthesized via the methylerythritol phosphate (MEP) pathway in plastids. During leaf maturation, curing, and processing, oxidative cleavage of carotenoids (e.g., β-carotene, neoxanthin) by carotenoid cleavage dioxygenases (CCDs) yields apocarotenoids such as β-damascone, megastigmatrienone, α-ionone, and dihydroactinidiolide (He et al., 2025). Neophytadiene, the most abundant terpenoid in tobacco, is a diterpene formed from the degradation of chlorophyll or via the terpenoid backbone pathway; it contributes to the characteristic “tobacco” note and is influenced by leaf maturity and curing conditions. The accumulation of these carotenoid-derived volatiles is strongly affected by light intensity, temperature, and nitrogen availability, which modulate carotenoid biosynthesis and degradation rates.

Phenylpropanoid/benzenoid compounds, including benzaldehyde, benzyl alcohol, p-tolualdehyde, and 2-methoxy-4-vinylphenol, are synthesized via the shikimate and phenylpropanoid pathways. Phenylalanine ammonia lyase (PAL) initiates the conversion of phenylalanine to cinnamic acid, which subsequently undergoes chain shortening, reduction, or methylation to yield various aromatic aldehydes and alcohols (Liu et al., 2026). 2-Methoxy-4-vinylphenol, a potent odorant with smoky and spicy notes, is a decarboxylation product of ferulic acid, a hydroxycinnamic acid derivative. These pathways are highly responsive to nitrogen nutrition and stress conditions, explaining the large variation observed under different fertilization regimes (**Table 4**).

Linalool is a monoterpene synthesized from geranyl diphosphate (GPP) via terpene synthases (TPS) in plastids (Li et al., 2021). Its fresh floral and woody character contributes to the green/woody dimension captured by PC2. Monoterpene biosynthesis is tightly regulated by developmental cues and environmental factors such as light and temperature, which may account for its relatively low variability across samples except under fertilization influence.

3-Hydroxy-2-butanone (acetoin) and related carbonyls arise from carbohydrate metabolism, particularly via glycolysis and the subsequent fermentation of pyruvate to acetoin by acetolactate synthase and acetolactate decarboxylase (Fincheira and Quiroz, 2018). These pathways are active during curing and are influenced by the redox status and microbial activity in the leaf tissue. 6-Methyl-5-hepten-2-ol is likely derived from the degradation of unsaturated fatty acids or from terpenoid metabolism, and its positive correlation with aroma quality suggests a role in enhancing fruity nuances.

The agronomic factors examined in this study—fertilization, harvesting method, plant growth vigor, and single-leaf weight—directly or indirectly modulate these biosynthetic pathways. For instance, nitrogen fertilization alters the carbon/nitrogen balance, affecting the flux through amino acid–derived pathways (e.g., phenylpropanoids) and potentially upregulating carotenoid biosynthesis at optimal nitrogen levels (Saenz et al., 2023). Harvesting method and timing influence the degree of tissue damage and the onset of curing-related enzymatic reactions, such as lipoxygenase-mediated fatty acid degradation and carotenoid cleavage. Mechanical harvest may accelerate cell disruption, promoting the formation of aldehydes and ketones that affect off-odor and irritation (Liang et al., 2021). Single-leaf weight, as an integrative measure of leaf thickness and dry matter accumulation, reflects the overall metabolic activity and sink strength, thereby correlating with the abundance of secondary metabolites (Dong et al., 2023).

Thus, the observed variation in key aroma components can be mechanistically linked to their biosynthetic origins and the regulatory effects of agronomic practices. This understanding not only validates the statistical correlations but also provides actionable targets for improving sensory quality through precision cultivation and processing. For tobacco quality control, we recommend establishing threshold values for the four VIP compounds (2-methoxy-4-vinylphenol, neophytadiene, scopoletin, and megastigmatrienone) identified through PLS-DA analysis as they can effectively discriminate regional characteristics and predict sensory profiles. Regarding agronomic practices, our findings indicate that nitrogen application timing should be optimized during the vegetative growth stage to enhance desirable compounds like 3-hydroxy-2-butanone and 2-methoxy-4-vinylphenol, while mechanical harvesting should be minimized for high-quality tobacco production since it significantly reduces carotenoid-derived volatiles that contribute to the characteristic sweet/fruity notes.

## 4 Conclusion

This study successfully established a sensory quality evaluation model applicable to major tobacco-producing regions in Henan through the systematic integration of sensory evaluation and chemical analysis. The research identified 13 key aroma components—including but not limited to 3-hydroxy-2-butanone, benzaldehyde, linalool, damascone and megastigmatrienone—that demonstrated significant correlations with sensory attributes such as aroma quality, off-odor, and commercial availability, underscoring their central role in the flavor profile of tobacco leaves. Principal component analysis further indicated that the aromatic characteristics of tobacco can be categorized into PC1, dominated by sweet and fruity notes, and PC2, characterized by green and woody attributes, providing a theoretical basis for the classification and targeted regulation of tobacco styles. By exploring the biosynthetic pathways of these key components-ranging from carotenoid degradation to phenylpropanoid and terpenoid metabolism-we gained insight into how agronomic factors modulate their accumulation. Fertilization was identified as the most influential factor affecting the variation in key aroma components, followed by harvesting method and single-leaf weight. Plant growth vigor showed limited impact on most components. These findings suggest that optimizing fertilization strategies and harvesting maturity can effectively modulate the composition and proportion of key aroma components in tobacco leaves through targeted metabolic regulation, thereby enhancing their sensory comfort and market competitiveness. The outcomes of this study not only deepen the understanding of the formation mechanism of sensory quality in Henan tobacco but also provide a feasible technical pathway and decision-making support for achieving “demand-oriented production” and “precision improvement” of tobacco leaves.

For immediate industry application, we propose a three-tiered quality control framework: 1) Field-level control: Monitor fertilization practices and harvesting methods to ensure optimal accumulation of the 13 key aroma components; 2) Processing-level control: Implement rapid GC-MS screening of VIP compounds (2-methoxy-4-vinylphenol, neophytadiene, scopoletin, megastigmatrienone) as quality indicators during curing and processing; 3) Product-level control: Use the established sensory-chemical correlation model to predict final product sensory performance and adjust blending ratios accordingly. This integrated approach bridges the gap between fundamental research and practical tobacco production, providing a scientific foundation for ‘demand-driven production’ and ‘precision improvement’ strategies in the tobacco industry.

It should be emphasized that the conclusions of this study are based on tobacco samples collected exclusively from three representative producing regions in Henan Province (Pingdingshan, Sanmenxia, and Nanyang). While the identified chemical-sensory relationships provide valuable insights for understanding the quality characteristics of Henan tobacco, the model’s generalizability to other geographical regions remains to be established. Future research should prioritize external validation of this model using independent datasets and samples from diverse tobacco-growing regions with varying climatic conditions, soil types, and cultivation practices. Such validation studies are essential to confirm the robustness of the 13 key aroma components as universal quality indicators and to assess whether the established sensory-chemical correlations hold across different tobacco varieties and production environments. Additionally, longitudinal studies tracking seasonal variations and multi-year datasets would further enhance the model’s reliability and practical utility for industry applications. Until such comprehensive validation is completed, caution should be exercised when extrapolating these findings to tobacco produced outside the studied regions, as regional-specific factors may significantly influence both chemical composition and sensory perception.

## Data Availability

The original contributions presented in the study are included in the article/**Supplementary Material**. Further inquiries can be directed to the corresponding authors.
