# Supplementary material for: Construction of a sensory quality evaluation model for tobacco leaves from Henan Province and its application in tobacco quality assessment
Source: Front Plant Sci. 2026 May 11;17:1792794. doi: 10.3389/fpls.2026.1792794 (PMC13199361; doi:10.3389/fpls.2026.1792794)
Supplement: Supplementary file 1 [file Table1.docx]

**Supporting Information**

**Construction of a sensory quality evaluation model for tobacco leaves from Henan Province and its application in tobacco quality assessment**

Xiujuan Xu^1^, Longhe Wang^3^, Jun Hu^1,*^, Xiaoyuan Tian^2^, Qingzhao Shi^1^, Kai Cui^1^, Xutao Li^2^, Weichen Zhang^1^, Shuoye Zhou^2^, Wenfen Zhang^3^, Guangqing Chen^2,*^, Chunqiang Yang^1,*^

*^1^ Zhengzhou Tobacco Research Institute of National Tobacco Corporation, Zhengzhou, China*

*^2^ Henan Tobacco Company of CNTC, Zhengzhou 450018, China*

*^3^ College of Chemistry, Zhengzhou University, Science Avenue #100, Zhengzhou, Henan, 450001, PR China.*

*Corresponding Authors: Jun Hu: [minsk998@126.com](mailto:minsk998@126.com), Guangqing Chen: [32779262@qq.com](mailto:32779262@qq.com), Chunqiang Yang: ycqflying2008@126.com

**Table S1.** Detailed information on the source of tobacco leaves.

| Sample | Region |
| --- | --- |
| 23L8108 | Pingdingshan |
| 23L8109 | Pingdingshan |
| 23L8114 | Pingdingshan |
| 23L8115 | Pingdingshan |
| 23L8119 | Pingdingshan |
| 23L8120 | Pingdingshan |
| 23L8123 | Pingdingshan |
| 23L8124 | Pingdingshan |
| 23L8128 | Pingdingshan |
| 23L8129 | Pingdingshan |
| 23L8131 | Pingdingshan |
| 23L8140 | Pingdingshan |
| 23L8136 | Pingdingshan |
| 23L8137 | Pingdingshan |
| 23L8145 | Pingdingshan |
| 23L8138 | Pingdingshan |
| 23L8144 | Pingdingshan |
| 23L8141 | Pingdingshan |
| 23L8139 | Pingdingshan |
| 23L8142 | Pingdingshan |
| L6313-1 | Sanmenxia |
| L6314-1 | Sanmenxia |
| L6324 | Sanmenxia |
| L6321 | Sanmenxia |
| L6315 | Sanmenxia |
| L6317 | Sanmenxia |
| L6316 | Sanmenxia |
| 23L8152 | Nanyang |
| 23L8156 | Nanyang |
| 23L8157 | Nanyang |
| 23L8165 | Nanyang |
| 23L8169 | Nanyang |
| 23L8172 | Nanyang |
| 23L8159 | Nanyang |
| 23L8162 | Nanyang |
| 23L8163 | Nanyang |
| 23L8164 | Nanyang |
| 23L8173 | Nanyang |
| 23L8174 | Nanyang |
| 23L8178 | Nanyang |
| 23L8181 | Nanyang |
| L6311-1 | Sanmenxia |
| L6311-2 | Sanmenxia |
| L6312-1 | Sanmenxia |
| L6312-2 | Sanmenxia |
| L6313-2 | Sanmenxia |
| L6314-2 | Sanmenxia |

**Table S2.** The retention times, quantitative ions and qualitative ions of nicotine and the internal standard.

| No. | Compound | RT/min | Quantitative ion（m/z） | Qualitative ion（m/z） | |
| --- | --- | --- | --- | --- | --- |
| 1 | Phenylethyl propionate (internal standard) | 14.823 | 122 | 104 | 178 |
| 2 | Nicotine | 17.044 | 84 | 133 | 161 |

**Table S3.** Detailed information on the quantitative analysis of 29 components in 47 tobacco leaf samples.

| Samples | 3-Hydroxy-2-butanone | Pentanol | 2,3-Butanediol | 2,3-Hexanedione | 6-Methyl-2-heptanol | Benzaldehyde | 6-Methyl-5-hepten-2-ol | Benzyl alcohol | Phenylacetaldehyde | Coumaran | p-Tolualdehyde | Linalool | Phenethyl alcohol | 2,3-Dihydro-3,5-dihydroxy-6-methyl-4(H)-pyran-4-one | 2,6,6-Trimethyl-2-cyclohexene-1,4-dione | 2-Methoxy-4-vinylphenol | Damascenone | α-Ionone | Dihydroactinidiolide | Megastigmatrienone | Ethyl laurate | Ethyl tetradecanoate | Neophytadiene | Farnesyl acetone | Scopoletin | Ethyl palmitate |
| --- | --- | --- | --- | --- | --- | --- | --- | --- | --- | --- | --- | --- | --- | --- | --- | --- | --- | --- | --- | --- | --- | --- | --- | --- | --- | --- |
| PDS-01 | 1.47 | 0.57 | 31.45 | 0.05 | 0.05 | 0.77 | 0.84 | 8.75 | 25.02 | 0 | 0.06 | 0.36 | 22.84 | 81.92 | 0.46 | 191.31 | 0.89 | 0.33 | 6.34 | 155.17 | 0.11 | 0.08 | 897.15 | 7.94 | 295.07 | 2.36 |
| PDS-02 | 1.04 | 0.99 | 31.12 | 0.11 | 0.07 | 0.94 | 1.24 | 12.26 | 33.54 | 0.07 | 0.07 | 0.41 | 36.33 | 64.52 | 0.54 | 176.81 | 1.06 | 0.32 | 6.43 | 176.65 | 0.11 | 0.09 | 937.37 | 9.44 | 335.28 | 2.33 |
| PDS-03 | 1.54 | 0.52 | 21.53 | 0.08 | 0.03 | 0.88 | 0.58 | 8.75 | 32.5 | 0.07 | 0.06 | 0.36 | 27.49 | 65.18 | 0.49 | 202.64 | 0.96 | 0.29 | 5.79 | 155.53 | 0.13 | 0.1 | 835.64 | 6.94 | 384.34 | 2.74 |
| PDS-04 | 1.08 | 0.44 | 18.25 | 0 | 0 | 0.81 | 0.81 | 9.05 | 28.78 | 0.07 | 0.07 | 0.35 | 25.06 | 81.97 | 0.47 | 269.11 | 0.95 | 0.3 | 6.25 | 154.45 | 0.11 | 0.11 | 756.45 | 6.37 | 434.49 | 3.18 |
| PDS-05 | 1.6 | 0.3 | 21.04 | 0.07 | 0.2 | 0.94 | 0.53 | 7.64 | 36.15 | 0.08 | 0.05 | 0.33 | 20.05 | 66.97 | 0.5 | 227.04 | 0.83 | 0.27 | 6.36 | 154.72 | 0.11 | 0.14 | 793.07 | 6.94 | 531.45 | 4.41 |
| PDS-06 | 1.48 | 0.71 | 25.23 | 0.05 | 0.03 | 0.81 | 0.91 | 9.61 | 29.62 | 0.07 | 0.09 | 0.35 | 30.53 | 47.79 | 0.51 | 314.65 | 0.99 | 0.31 | 6.31 | 173.6 | 0.11 | 0.13 | 904.9 | 7.97 | 541.39 | 5.02 |
| PDS-07 | 2.11 | 0.97 | 24.19 | 0.07 | 0.03 | 0.93 | 0.73 | 13.97 | 26.98 | 0.07 | 0.06 | 0.36 | 42.28 | 40.62 | 0.58 | 189.74 | 0.8 | 0.27 | 5.9 | 176.68 | 0.11 | 0.14 | 720.29 | 7.26 | 386.3 | 4.84 |
| PDS-08 | 1.48 | 0.45 | 18.72 | 0 | 0.07 | 0.95 | 1.2 | 11.08 | 39.53 | 0.07 | 0.08 | 0.35 | 32.57 | 27.56 | 0.49 | 365.35 | 0.95 | 0.28 | 6.35 | 170.39 | 0.11 | 0.15 | 901.66 | 6.66 | 467.37 | 6.05 |
| PDS-09 | 2.24 | 0.66 | 14.65 | 0 | 0 | 0.93 | 0.99 | 12.52 | 22.61 | 0.07 | 0.08 | 0.38 | 29.38 | 15.39 | 0.54 | 346.25 | 1.17 | 0.34 | 7.45 | 180.08 | 0.11 | 0.18 | 833.42 | 8.39 | 695.24 | 5.01 |
| PDS-10 | 1.96 | 0.68 | 16.16 | 0 | 0.09 | 1.14 | 1.29 | 12.89 | 40.6 | 0.07 | 0.08 | 0.39 | 38.82 | 13.89 | 0.51 | 368.89 | 1.11 | 0.32 | 6.18 | 194.72 | 0.11 | 0.15 | 824.26 | 6.21 | 410.62 | 4.56 |
| PDS-11 | 3.63 | 0.26 | 16.86 | 0.24 | 0.04 | 0.94 | 1.28 | 17.93 | 16.8 | 0.07 | 0.08 | 0.43 | 22.94 | 10.23 | 0.57 | 632.22 | 1.58 | 0.3 | 5.83 | 246.58 | 0.11 | 0.19 | 1113.72 | 7.43 | 1124.95 | 4.74 |
| PDS-12 | 5.46 | 0.29 | 28.17 | 0 | 0 | 0.71 | 1.28 | 17.24 | 16.05 | 0.07 | 0.07 | 0.32 | 23.51 | 38.17 | 0.86 | 313.05 | 1.02 | 0.3 | 6.46 | 193.05 | 0 | 0.13 | 791.75 | 9.26 | 466.57 | 3.47 |
| PDS-13 | 5.08 | 0.54 | 33.12 | 0 | 0 | 0.78 | 0.97 | 17.04 | 10.7 | 0.07 | 0.07 | 0.34 | 21.8 | 29.64 | 0.75 | 290.45 | 1.06 | 0.29 | 6.27 | 179.4 | 0 | 0.13 | 952.23 | 8.59 | 502.38 | 2.74 |
| PDS-14 | 2.45 | 0.37 | 17.19 | 0 | 0 | 0.69 | 0.88 | 16.31 | 9.04 | 0.07 | 0.06 | 0.33 | 14.1 | 21.86 | 0.58 | 323.32 | 1.02 | 0.3 | 7.76 | 157.07 | 0 | 0.1 | 1051.73 | 7.63 | 741.81 | 2.23 |
| PDS-15 | 3.95 | 0.59 | 25.16 | 0 | 0 | 0.98 | 0.89 | 17.89 | 21.81 | 0.07 | 0.06 | 0.37 | 29.23 | 40.9 | 0.56 | 272.43 | 1.15 | 0.3 | 6.15 | 208.89 | 0 | 0.12 | 1096.69 | 7.81 | 456.38 | 2.73 |
| PDS-16 | 4.86 | 0.47 | 28.06 | 0 | 0 | 0.74 | 0.89 | 17.59 | 13.07 | 0.07 | 0.06 | 0.35 | 25.48 | 43.34 | 0.54 | 307.6 | 1.14 | 0.31 | 6.45 | 199.49 | 0 | 0.18 | 1028.02 | 8.3 | 586.56 | 4.67 |
| PDS-17 | 2.55 | 0.6 | 19.19 | 0 | 0 | 0.69 | 1.17 | 20.85 | 9.24 | 0.07 | 0.06 | 0.36 | 21.96 | 20.7 | 0.55 | 361.71 | 1.17 | 0.3 | 7.22 | 183.75 | 0 | 0.13 | 895.54 | 9.06 | 817.4 | 3.19 |
| PDS-18 | 3.62 | 0.7 | 24.75 | 0 | 0 | 0.82 | 0.76 | 13.86 | 14.4 | 0.08 | 0.06 | 0.37 | 25.77 | 36.71 | 0.56 | 273.59 | 1.02 | 0.3 | 7.66 | 172.74 | 0 | 0.34 | 951.87 | 8.85 | 541.13 | 10.08 |
| PDS-19 | 2.58 | 0.69 | 21.17 | 0 | 0 | 0.76 | 0.97 | 19.11 | 12.38 | 0.08 | 0.06 | 0.39 | 22.56 | 15.45 | 0.63 | 366.47 | 1.16 | 0.31 | 7.33 | 178.36 | 0 | 0.55 | 960.72 | 9.15 | 722.28 | 7.73 |
| PDS-20 | 2.37 | 1.05 | 20.74 | 0 | 0 | 0.77 | 0.85 | 21.42 | 8.97 | 0.08 | 0.06 | 0.39 | 23.75 | 18.69 | 0.71 | 347.45 | 1.07 | 0.32 | 8.05 | 158.33 | 0.15 | 1.23 | 820.61 | 9.54 | 655.97 | 11.68 |
| SMX-01 | 3.22 | 0.31 | 16.94 | 0 | 0 | 0.74 | 0.93 | 17.97 | 22.1 | 0.07 | 0.07 | 0.34 | 11.91 | 21.59 | 0.49 | 590.56 | 1.35 | 0.33 | 7.47 | 156.66 | 0 | 0.19 | 830.25 | 8.34 | 378.47 | 3.95 |
| SMX-02 | 3.33 | 0.23 | 15.51 | 0 | 0 | 0.63 | 0.99 | 17.94 | 11.66 | 0.07 | 0.08 | 0.33 | 11.2 | 36.06 | 0.51 | 639.73 | 1.25 | 0.35 | 7.79 | 144.19 | 0 | 0.14 | 728.2 | 8.38 | 418.47 | 3.47 |
| SMX-03 | 3.35 | 0.7 | 15.86 | 0 | 0 | 0.85 | 0.93 | 23.39 | 19.95 | 0.08 | 0.07 | 0.38 | 17.03 | 26.29 | 0.55 | 509.17 | 1.49 | 0.36 | 7.65 | 167.53 | 0 | 0.2 | 882 | 9.46 | 371.07 | 3.87 |
| SMX-04 | 3.99 | 0.15 | 11.71 | 0 | 0 | 0.67 | 1.21 | 17.92 | 11.65 | 0.07 | 0.07 | 0.3 | 10.12 | 33.45 | 0.39 | 778 | 1.13 | 0.36 | 8.02 | 149.31 | 0 | 0.17 | 592.04 | 9.46 | 198.11 | 4.52 |
| SMX-05 | 3.77 | 0 | 12.25 | 0 | 0 | 0.6 | 1.15 | 13.81 | 17.49 | 0.08 | 0.07 | 0.32 | 8.2 | 44.62 | 0.3 | 964.83 | 1.01 | 0.36 | 7.68 | 147.47 | 0 | 0.15 | 795.33 | 6.09 | 248.91 | 4.2 |
| SMX-06 | 4.26 | 0.09 | 11.85 | 0 | 0 | 0.87 | 0.83 | 13.73 | 21.1 | 0.08 | 0.07 | 0.32 | 9.35 | 23.92 | 0.42 | 672.69 | 1.2 | 0.35 | 8.53 | 190.23 | 0 | 0.33 | 1133.12 | 8.92 | 138.27 | 8.45 |
| SMX-07 | 4.38 | 0 | 12.54 | 0 | 0 | 0.74 | 0.96 | 13.62 | 15.7 | 0.08 | 0.07 | 0.31 | 8.72 | 22.84 | 0.39 | 735.21 | 1.15 | 0.34 | 7.38 | 184.12 | 0 | 0.15 | 1058.19 | 7.68 | 132.98 | 3.02 |
| SMX-08 | 2.62 | 0.07 | 12.88 | 0 | 0 | 0.56 | 0.88 | 14.61 | 12.58 | 0.08 | 0.06 | 0.32 | 10.34 | 55.78 | 0.46 | 670.87 | 1.26 | 0.32 | 7 | 145.11 | 0 | 0.13 | 723.9 | 8.29 | 273.81 | 2.17 |
| SMX-09 | 2.73 | 0.04 | 12.43 | 0 | 0 | 0.55 | 0.8 | 14.6 | 11.45 | 0.08 | 0.06 | 0.31 | 9.93 | 60.79 | 0.4 | 703.51 | 1.27 | 0.33 | 7.28 | 144.92 | 0 | 0.14 | 834.41 | 7.76 | 280.16 | 2.23 |
| SMX-10 | 2.59 | 0.09 | 16.22 | 0 | 0 | 0.51 | 0.95 | 12.91 | 8.66 | 0.08 | 0.07 | 0.31 | 9.35 | 52.54 | 0.38 | 839.24 | 1.1 | 0.36 | 8.11 | 129.48 | 0 | 0.11 | 570.06 | 8.24 | 382.28 | 2.63 |
| SMX-11 | 2.54 | 0.08 | 15.62 | 0 | 0 | 0.53 | 0.91 | 15.15 | 10.73 | 0.08 | 0.07 | 0.32 | 10.38 | 67.32 | 0.36 | 823.08 | 1.15 | 0.37 | 8.05 | 129.68 | 0 | 0.13 | 604.98 | 7.91 | 398.58 | 2.64 |
| SMX-12 | 2.48 | 0.1 | 15.23 | 0 | 0 | 0.6 | 0.78 | 15.12 | 12.37 | 0.08 | 0.06 | 0.32 | 10.49 | 39.85 | 0.38 | 633.78 | 1.31 | 0.34 | 8.27 | 146.08 | 0 | 0.11 | 800.36 | 8.19 | 251.64 | 2.33 |
| SMX-13 | 2.46 | 0.12 | 14.73 | 0 | 0 | 0.51 | 0.88 | 13.98 | 9.63 | 0.07 | 0.06 | 0.3 | 9.86 | 40.8 | 0.34 | 601.08 | 1.23 | 0.35 | 7.57 | 142.67 | 0 | 0.13 | 680.72 | 9.19 | 199.73 | 2.6 |
| NY-01 | 3.81 | 0.37 | 18.25 | 0 | 0 | 0.72 | 1.16 | 17.93 | 17.57 | 0.08 | 0.07 | 0.34 | 14.64 | 41.68 | 0.53 | 434.16 | 1.33 | 0.34 | 7.83 | 185.11 | 0 | 0.11 | 1309.86 | 6.98 | 283.34 | 3.17 |
| NY-02 | 4.6 | 0.31 | 21.52 | 0 | 0 | 0.69 | 1.02 | 16.5 | 15.82 | 0.08 | 0.07 | 0.34 | 12.63 | 35.55 | 0.56 | 366.67 | 1.37 | 0.35 | 7.58 | 185.51 | 0 | 0.11 | 1296.29 | 7.31 | 265.54 | 2.91 |
| NY-03 | 4.35 | 0.51 | 22.69 | 0 | 0 | 0.73 | 0.99 | 17.17 | 14.8 | 0.08 | 0.07 | 0.33 | 15.21 | 29.19 | 0.52 | 379.73 | 1.32 | 0.33 | 7.8 | 178.69 | 0 | 0.12 | 1111.1 | 8.36 | 238.95 | 3.24 |
| NY-04 | 4.44 | 0.98 | 20.89 | 0 | 0 | 0.84 | 1.94 | 27.75 | 16.72 | 0.08 | 0.08 | 0.39 | 20.6 | 21.92 | 0.71 | 494.9 | 1.69 | 0.35 | 8.44 | 208.55 | 0 | 0.12 | 1140.45 | 10.47 | 368.9 | 2.76 |
| NY-05 | 5.21 | 1.21 | 22.59 | 0 | 0 | 0.91 | 1.5 | 26.27 | 19.8 | 0.08 | 0.08 | 0.37 | 22.38 | 27.83 | 0.71 | 403.23 | 1.64 | 0.35 | 8.33 | 222.62 | 0 | 0.14 | 1143.36 | 11.71 | 305.29 | 2.81 |
| NY-06 | 4.48 | 0.78 | 19.95 | 0 | 0 | 0.85 | 1.07 | 22.64 | 16.27 | 0.08 | 0.07 | 0.39 | 20.42 | 23.41 | 0.68 | 383.18 | 1.52 | 0.35 | 8.07 | 197.66 | 0 | 0.12 | 1033.37 | 11.87 | 264.77 | 2.71 |
| NY-07 | 4.81 | 0.08 | 25.8 | 0 | 0 | 0.66 | 0.92 | 13.08 | 13.46 | 0.08 | 0.08 | 0.32 | 10.49 | 31.46 | 0.56 | 843.1 | 1.26 | 0.35 | 9.82 | 196.86 | 0 | 0.13 | 1139.19 | 10.86 | 158.63 | 2.44 |
| NY-08 | 3.68 | 0.31 | 20.68 | 0 | 0 | 0.73 | 1.15 | 17.71 | 13.72 | 0.08 | 0.07 | 0.33 | 14.63 | 39.15 | 0.79 | 353.39 | 1.37 | 0.34 | 10.79 | 200.52 | 0 | 0.08 | 1106.41 | 16.73 | 250.34 | 2.49 |
| NY-09 | 3.53 | 0.29 | 19.87 | 0 | 0 | 0.75 | 0.93 | 17.28 | 14.29 | 0.08 | 0.08 | 0.33 | 15.2 | 22.45 | 0.75 | 385.85 | 1.46 | 0.35 | 10.8 | 214.03 | 0 | 0.11 | 1132.96 | 17.58 | 248.33 | 2.79 |
| NY-10 | 3.8 | 0.55 | 18.82 | 0 | 0 | 0.77 | 0.98 | 18.27 | 19.71 | 0.08 | 0.07 | 0.34 | 15.81 | 25.87 | 0.68 | 339.08 | 1.46 | 0.36 | 10.59 | 206.95 | 0 | 0.11 | 1049.77 | 16.17 | 278.55 | 2.68 |
| NY-11 | 3.64 | 0.82 | 20.69 | 0 | 0 | 0.67 | 1.81 | 21.18 | 9.14 | 0.08 | 0.07 | 0.36 | 18.72 | 44.65 | 0.93 | 546.91 | 1.46 | 0.37 | 9.56 | 201.66 | 0 | 0.12 | 984.53 | 15.55 | 345.95 | 2.7 |
| NY-12 | 3.27 | 0.69 | 17.83 | 0 | 0 | 0.73 | 1.11 | 19.26 | 10.03 | 0.08 | 0.07 | 0.37 | 17.61 | 31.69 | 0.75 | 479.04 | 1.69 | 0.41 | 8.81 | 213.02 | 0 | 0.14 | 1073.88 | 12.76 | 395.9 | 2.59 |
| NY-13 | 3.38 | 0.57 | 18.67 | 0 | 0 | 0.8 | 1.02 | 19.22 | 11.98 | 0.08 | 0.06 | 0.37 | 17.39 | 20.62 | 0.67 | 415.81 | 1.61 | 0.38 | 8.46 | 200.4 | 0 | 0.15 | 1116.63 | 12.07 | 464.58 | 2.66 |
| NY-14 | 4.02 | 0.52 | 21.17 | 0 | 0 | 0.65 | 2.04 | 18.36 | 7.99 | 0.08 | 0.08 | 0.35 | 15.42 | 39.55 | 0.82 | 551.13 | 1.52 | 0.35 | 9.64 | 205.42 | 0 | 0.11 | 1059.98 | 15.49 | 375.09 | 2.64 |

**Table S4.** The minimum, maximum, average, standard deviation and coefficient of variation of the contents of 28 components in 47 samples.

| Compound | Minimum  (μg g^-1^) | Maximum  (μg g^-1^) | Average  (μg g^-1^) | Standard deviation | Coefficient of variation/% |
| --- | --- | --- | --- | --- | --- |
| Nicotine | 7310 | 23790 | 17670 | 3950 | 22.37 |
| 3-Hydroxy-2-butanone | 1.04 | 5.46 | 3.31 | 1.22 | 36.79 |
| Pentanol | 0.00 | 1.21 | 0.52 | 0.29 | 55.44 |
| 2,3-Butanediol | 11.71 | 33.12 | 20.56 | 5.18 | 25.20 |
| 2,3-Hexanedione | 0.00 | 0.24 | 0.02 | 0.04 | 273.58 |
| 6-Methyl-2-heptanol | 0.00 | 0.20 | 0.01 | 0.04 | 250.64 |
| Benzaldehyde | 0.60 | 1.14 | 0.79 | 0.11 | 14.36 |
| 6-Methyl-5-hepten-2-ol | 0.53 | 2.04 | 1.07 | 0.31 | 29.32 |
| Benzyl alcohol | 7.64 | 27.75 | 16.51 | 4.59 | 27.82 |
| Phenylacetaldehyde | 7.99 | 40.60 | 18.75 | 8.64 | 46.10 |
| Coumaran | 0.00 | 0.08 | 0.07 | 0.01 | 17.37 |
| p-Tolualdehyde | 0.05 | 0.09 | 0.07 | 0.01 | 12.20 |
| Linalool | 0.30 | 0.43 | 0.35 | 0.03 | 8.01 |
| Phenethyl alcohol | 8.20 | 42.28 | 20.59 | 8.24 | 40.04 |
| 2,3-Dihydro-3,5-dihydroxy-6-methyl-4(H)-pyran-4-one | 10.23 | 81.97 | 34.76 | 17.13 | 49.27 |
| 2,6,6-Trimethyl-2-cyclohexene-1,4-dione | 0.30 | 0.93 | 0.59 | 0.14 | 22.98 |
| 2-Methoxy-4-vinylphenol | 176.81 | 964.83 | 424.55 | 184.15 | 43.37 |
| Damascenone | 0.80 | 1.69 | 1.23 | 0.24 | 19.92 |
| α-Ionone | 0.27 | 0.41 | 0.33 | 0.03 | 9.46 |
| Dihydroactinidiolide | 5.79 | 10.80 | 7.65 | 1.37 | 17.89 |
| Megastigmatrienone | 144.19 | 246.58 | 183.56 | 23.22 | 12.65 |
| Ethyl laurate | 0.00 | 0.15 | 0.03 | 0.05 | 158.57 |
| Ethyl tetradecanoate | 0.08 | 1.23 | 0.18 | 0.19 | 104.13 |
| Neophytadiene | 592.04 | 1309.86 | 970.34 | 161.16 | 16.61 |
| Farnesyl acetone | 6.09 | 17.58 | 9.56 | 3.01 | 31.46 |
| Scopoletin | 132.98 | 1124.95 | 420.18 | 200.31 | 47.67 |
| Ethyl palmitate | 2.23 | 11.68 | 4.00 | 2.10 | 52.64 |

**Table S5.** VIP values of 26 compounds.

| **Var ID (Primary)** | **VIP** |
| --- | --- |
| 2-Methoxy-4-vinylphenol | 2.9978 |
| Neophytadiene | 2.85661 |
| Scopoletin | 2.56711 |
| Megastigmatrienone | 1.02732 |
| Phenethyl alcohol | 0.633421 |
| 2,3-Butanediol | 0.406384 |
| Phenylacetaldehyde | 0.386097 |
| Farnesyl acetone | 0.356133 |
| Benzyl alcohol | 0.326397 |
| 2,3-Dihydro-3,5-dihydroxy-6-methyl-4(H)-pyran-4-one | 0.295419 |
| Dihydroactinidiolide | 0.266117 |
| Ethyl palmitate | 0.170199 |
| 3-Hydroxy-2-butanone | 0.168464 |
| Damascenone | 0.113031 |
| Pentanol | 0.0915614 |
| 6-Methyl-5-hepten-2-ol | 0.0782049 |
| 2,6,6-Trimethyl-2-cyclohexene-1,4-dione | 0.0781327 |
| Benzaldehyde | 0.060056 |
| Ethyl laurate | 0.0425672 |
| α-Ionone | 0.0401025 |
| Ethyl tetradecanoate | 0.0295779 |
| Linalool | 0.0266227 |
| 6-Methyl-2-heptanol | 0.0226936 |
| 2,3-Hexanedione | 0.0225259 |
| Coumaran | 0.0135045 |
| p-Tolualdehyde | 0.00831131 |

To support the analysis of key groups related to the sensory quality of tobacco leaves in central Henan, this section provides detailed experimental procedures and data results. Two tobacco samples, L140 and L109, with significant differences in sensory quality from the central Henan production area, were selected for this study. After obtaining tobacco extracts through aqueous extraction, the extracts were separated via column chromatography using Sephadex LH-20. This process yielded 16 fractions from L140 and 15 from L109 (31 fractions in total, divided into 17 comparative evaluation groups). Each fraction was evaluated through sensory assessment and scored (**Table S6**). Based on the sensory results, representative high-quality fractions (L140-8 and L109-8) and poor-quality fractions (L140-7 and L109-7) were randomly selected for subsequent chemical composition analysis.

Headspace solid-phase microextraction combined with gas chromatography-mass spectrometry (HS-SPME-GC-MS) was employed to analyze the volatile components in the four fractions. A total of 91 compounds were identified, with 80, 62, 62, and 50 compounds detected in L140-8, L109-8, L109-7, and L140-7, respectively. After verification with standard compounds, 29 key compounds were confirmed (**Table 1**).

**Table S6 Sensory evaluation results of separated components.**

| **Group** | **Separation component number** | **Quality of aroma** | **Volume of aroma** | **Offensive taste** | **concentration** | **Strength** | **Aroma release intensity** | **Smooth** | **Mellow** | **Sweet aftertaste** | **Irritancy** | **Residual sweetness** | **Hay like** | **Burnt sweet** | **Mellow sweet** | **Fresh-sweet** | **Burnt aroma** |
| --- | --- | --- | --- | --- | --- | --- | --- | --- | --- | --- | --- | --- | --- | --- | --- | --- | --- |
| 1 | L140-0 | 18.0 | 18.0 | 12.0 | 6.0 | 0.0 | 12.0 | 12.0 | 6.0 | 12.0 | 0.0 | 0.0 | 0.0 | 8.4 | 12.0 | 0.0 | 0.0 |
|  | L109-0 | 0.0 | 6.0 | 6.0 | 12.0 | 0.0 | 0.0 | 0.0 | 0.0 | 6.0 | 0.0 | 0.0 | 0.0 | 6.0 | 0.0 | 0.0 | 0.0 |
| 2 | L140-1 | 6.0 | 12.0 | 6.0 | 6.0 | 0.0 | 6.0 | 6.0 | 0.0 | 6.0 | 0.0 | 0.0 | 0.0 | 6.0 | 6.0 | 0.0 | 0.0 |
|  | L109-1 | 0.0 | 0.0 | -6.0 | 0.0 | 0.0 | 0.0 | 6.0 | 0.0 | 0.0 | -6.0 | -2.4 | 0.0 | 0.0 | 0.0 | 0.0 | 6.0 |
| 3 | L140-2 | 0.0 | 6.0 | 0.0 | 0.0 | 0.0 | 0.0 | 6.0 | 0.0 | 6.0 | 0.0 | 0.0 | 0.0 | 6.0 | 0.0 | 0.0 | 0.0 |
|  | L109-2 | -6.0 | 0.0 | -6.0 | 0.0 | 0.0 | 0.0 | 0.0 | 0.0 | 0.0 | -7.2 | -6.0 | 0.0 | 0.0 | 0.0 | 0.0 | 12.0 |
| 4 | L140-1+L140-2 | 6.0 | 6.0 | 0.0 | 0.0 | 0.0 | 0.0 | 6.0 | 0.0 | 6.0 | 0.0 | 0.0 | 0.0 | 6.0 | 6.0 | 0.0 | 0.0 |
|  | L109-1+L140-2 | 0.0 | 6.0 | -6.0 | 0.0 | 0.0 | 0.0 | 6.0 | 0.0 | 6.0 | -6.0 | 0.0 | 0.0 | 0.0 | 0.0 | 0.0 | 6.0 |
| 5 | L140-3 | 12.0 | 18.0 | 12.0 | 6.0 | 0.0 | 6.0 | 12.0 | 6.0 | 12.0 | 0.0 | 0.0 | 0.0 | 6.0 | 12.0 | 0.0 | 0.0 |
|  | L109-3 | 6.0 | 9.6 | 6.0 | 0.0 | 0.0 | 6.0 | 6.0 | 0.0 | 6.0 | 0.0 | 0.0 | 0.0 | 6.0 | 0.0 | 0.0 | 0.0 |
| 6 | L140-4 | 0.0 | 6.0 | 6.0 | 0.0 | 0.0 | 0.0 | 6.0 | 0.0 | 0.0 | 0.0 | -6.0 | 0.0 | 0.0 | 0.0 | 0.0 | 0.0 |
|  | L109-4 | -12.0 | -18.0 | -12.0 | 0.0 | 0.0 | 0.0 | 0.0 | 0.0 | 0.0 | -12.0 | -12.0 | 0.0 | 0.0 | 0.0 | 0.0 | 0.0 |
| 7 | L140-5 | -6.0 | 0.0 | -6.0 | 0.0 | 0.0 | 0.0 | 6.0 | 0.0 | 0.0 | 0.0 | -6.0 | 0.0 | 6.0 | 0.0 | 0.0 | 0.0 |
|  | L109-5 | -18.0 | -18.0 | -18.0 | 0.0 | 0.0 | 0.0 | 0.0 | 0.0 | 0.0 | -12.0 | -12.0 | 0.0 | 0.0 | 0.0 | 0.0 | 6.0 |
| 8 | L140-4+L140-5 | 0.0 | 6.0 | 6.0 | 0.0 | 0.0 | 0.0 | 6.0 | 0.0 | 0.0 | 0.0 | 0.0 | 0.0 | 0.0 | 0.0 | 0.0 | 6.0 |
|  | L109-4+L109-5 | -6.0 | -6.0 | -6.0 | 0.0 | 0.0 | 0.0 | 0.0 | 0.0 | 0.0 | 0.0 | -9.6 | 0.0 | 0.0 | 0.0 | 0.0 | 0.0 |
| 9 | L140-6 | 6.0 | 12.0 | 6.0 | 0.0 | 0.0 | 6.0 | 6.0 | 0.0 | 6.0 | 0.0 | 0.0 | 0.0 | 6.0 | 6.0 | 0.0 | 0.0 |
|  | L109-6 | 0.0 | 6.0 | -6.0 | 0.0 | 0.0 | 6.0 | 6.0 | 0.0 | 0.0 | 0.0 | 0.0 | 0.0 | 0.0 | 0.0 | 0.0 | 6.0 |
| 10 | L140-7 | -6.0 | 0.0 | -6.0 | 0.0 | 0.0 | 0.0 | 0.0 | 0.0 | 0.0 | -6.0 | 0.0 | 0.0 | 0.0 | 0.0 | 0.0 | 0.0 |
|  | L109-7 | -24.0 | -18.0 | -18.0 | 0.0 | 0.0 | 0.0 | 0.0 | 0.0 | 0.0 | -18.0 | -18.0 | 0.0 | 0.0 | 0.0 | 0.0 | 4.8 |
| 11 | L140-8 | 18.0 | 18.0 | 12.0 | 6.0 | 0.0 | 12.0 | 12.0 | 12.0 | 12.0 | 0.0 | 0.0 | 0.0 | 12.0 | 12.0 | 0.0 | 0.0 |
|  | L109-8 | 6.0 | 12.0 | 6.0 | 6.0 | 0.0 | 6.0 | 0.0 | 0.0 | 0.0 | -5.3 | 0.0 | 0.0 | 6.0 | 0.0 | 0.0 | 6.0 |
| 12 | L140-9 | 0.0 | 6.0 | 0.0 | 0.0 | 0.0 | 0.0 | 0.0 | 0.0 | 4.5 | -4.5 | -1.5 | 0.0 | 0.0 | 0.0 | 0.0 | 6.0 |
|  | L109-9 | 0.0 | 6.0 | -12.0 | 0.0 | 0.0 | 0.0 | 0.0 | 0.0 | -0.8 | -5.3 | -5.3 | -0.8 | 0.0 | 0.0 | 0.0 | 11.3 |
| 13 | L140-10 | 0.0 | -6.0 | 0.0 | 0.0 | 0.0 | 0.0 | 6.0 | 0.0 | 3.0 | 0.0 | 0.0 | 0.0 | 0.0 | 0.0 | 0.0 | 0.0 |
|  | L109-10 | -12.0 | 0.0 | -12.0 | 0.0 | 0.0 | 0.0 | 0.0 | 0.0 | 0.0 | -2.3 | -6.8 | 0.0 | 1.5 | 0.0 | 0.0 | 10.5 |
| 14 | L140-11 | 0.0 | 0.0 | -6.0 | 0.0 | 0.0 | 0.0 | 6.0 | 0.0 | 0.0 | -1.5 | 0.0 | 0.0 | 0.0 | 0.0 | 0.0 | 0.0 |
|  | L109-11 | -12.0 | 0.0 | -12.0 | 0.0 | 0.0 | 0.0 | 0.0 | 0.0 | 0.0 | -9.8 | -8.3 | 0.0 | 0.0 | 0.0 | 0.0 | 9.0 |
| 15 | L140-10+L140-11 | 0.0 | 0.0 | -12.0 | 0.0 | 0.0 | 0.0 | 0.0 | 0.0 | 0.0 | -6.0 | -12.0 | 0.0 | 0.0 | 0.0 | 0.0 | 12.0 |
|  | L109-10+L109-11 | 0.0 | 0.0 | -18.0 | 0.0 | 0.0 | 0.0 | 0.0 | 0.0 | 0.0 | -6.0 | -12.0 | 0.0 | 0.0 | 0.0 | 0.0 | 6.0 |
| 16 | L140-12 | 0.0 | 0.0 | 0.0 | 0.0 | -1.5 | 6.0 | 0.0 | 0.0 | 0.0 | -6.0 | -1.5 | 0.0 | 0.0 | 0.0 | 0.0 | 0.0 |
|  | L109-12 | 0.0 | 0.0 | 0.0 | 0.0 | 0.0 | 0.0 | 6.0 | 0.0 | 6.0 | 0.0 | -1.5 | 0.0 | 0.0 | 0.0 | 0.0 | 6.0 |
|  | L140-13 | 0.0 | 0.0 | 0.0 | 0.0 | 0.0 | 0.0 | 6.0 | 0.0 | 6.8 | 0.0 | 0.0 | 0.0 | 6.0 | 0.0 | 0.0 | 0.0 |
|  | L140-14 | -6.0 | 0.0 | -6.0 | 0.0 | 0.0 | 0.0 | 0.0 | 0.0 | 0.0 | -9.0 | -5.3 | 0.0 | 0.0 | 0.0 | 0.0 | 6.0 |
| 17 | L140-15 | -6.0 | 0.0 | 0.0 | 0.0 | 0.0 | 0.0 | 6.0 | 0.0 | 0.0 | -6.0 | 0.0 | 0.0 | 6.0 | 0.0 | 0.0 | 0.0 |
|  | L140-16 | 0.0 | 0.0 | 0.0 | 0.0 | 0.0 | 0.0 | 0.0 | 0.0 | 0.0 | -6.0 | 0.0 | 0.0 | 0.0 | 0.0 | 0.0 | 6.0 |
|  | L109-13 | 0.0 | 0.0 | -6.0 | 0.0 | 0.0 | 0.0 | 0.0 | 0.0 | 0.0 | 0.0 | -6.0 | 0.0 | 0.0 | 0.0 | 0.0 | 12.0 |
|  | L109-14 | 0.0 | 0.0 | -6.0 | 0.0 | 0.0 | 0.0 | 0.0 | 0.0 | 0.0 | -12.0 | -12.0 | 0.0 | 0.0 | 0.0 | 0.0 | 6.0 |
|  | L109-15 | 0.0 | 0.0 | 0.0 | 0.0 | 0.0 | 0.0 | 0.0 | 0.0 | 0.0 | -6.0 | -6.0 | 0.0 | 0.0 | 0.0 | 0.0 | 2.3 |
